# Supplementary material for: Hachiman is a genome integrity sensor
Source: bioRxiv. 2024 Feb 29:2024.02.29.582594. Preprint. [Version 1] doi: 10.1101/2024.02.29.582594 (PMC10925250; doi:10.1101/2024.02.29.582594)
Supplement: Supplement 1 [file media-1.pdf]

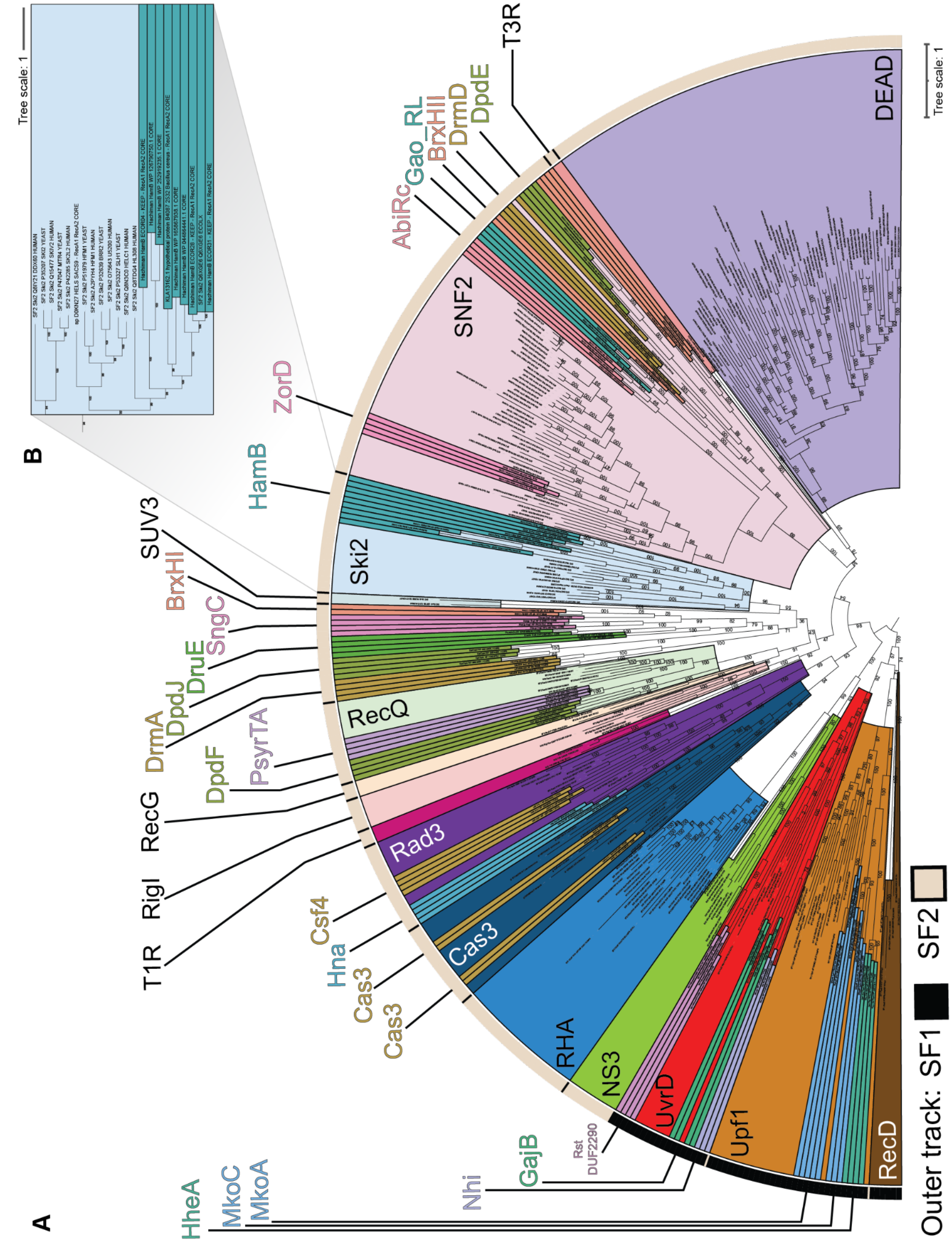

Figure S1 | Helicases and Hachiman phylogenetic analysis. **(A)** Annotated phylogenetic tree of phage defense system associated helicase core domains and reference helicases shown in Fig. 1B. Bootstrapping values determined by UFBoot2[72] are shown. **(B)** Zoomed-in view focusing on represented Ski2 helicases.

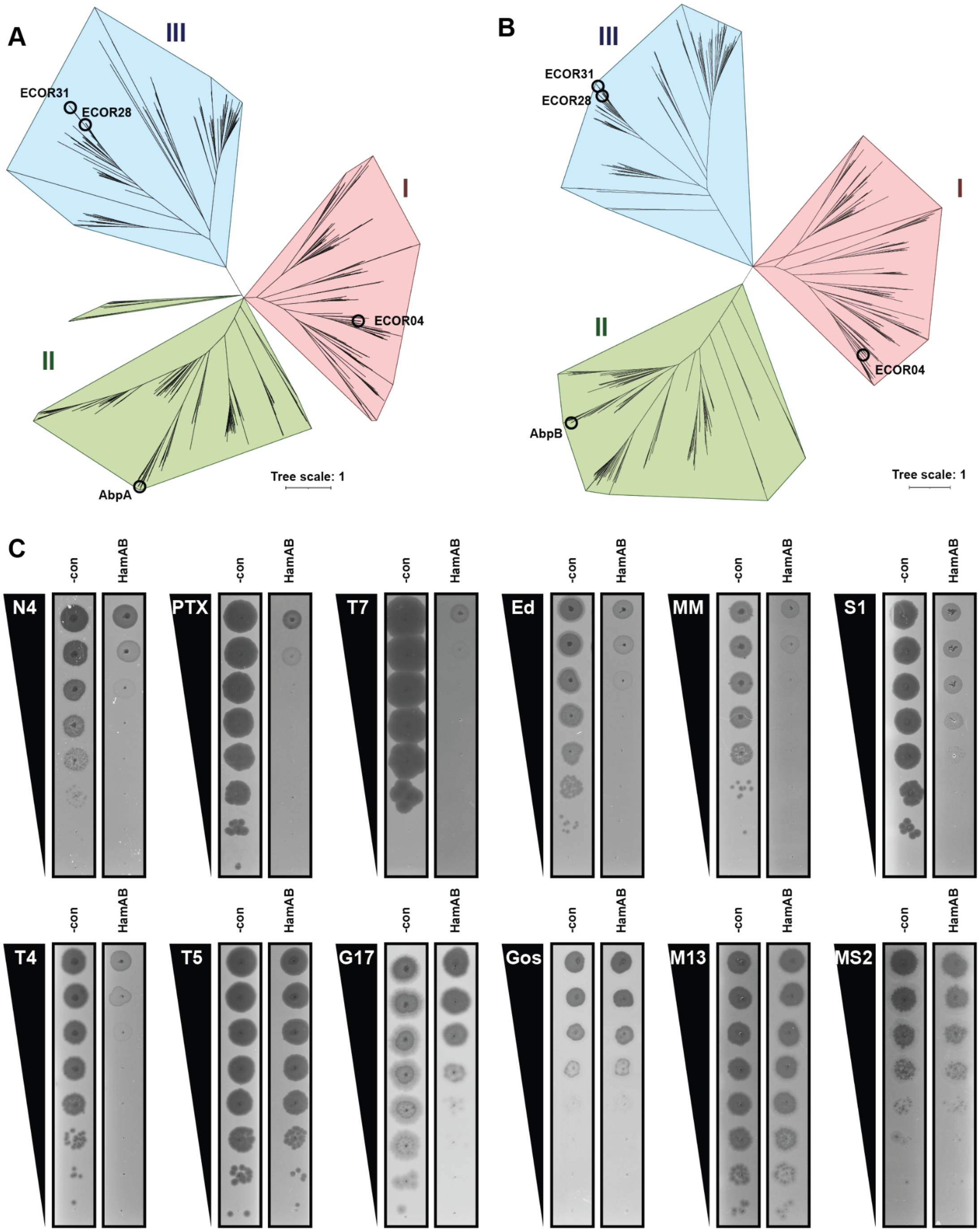

Figure S2 | Function and phylogeny of tested HamAB proteins. **(A, B)** Phylogenetic tree of (A) HamA and (B) HamB from DefenseFinder[17] with HamA and HamB sequences from this manuscript and AbpAB[34] are labeled. We assign 3 potential clades of HamB and their corresponding HamA clades as I-III. **(C)** Representative plaque assays for ECOR31 HamAB and phages tested in this study.

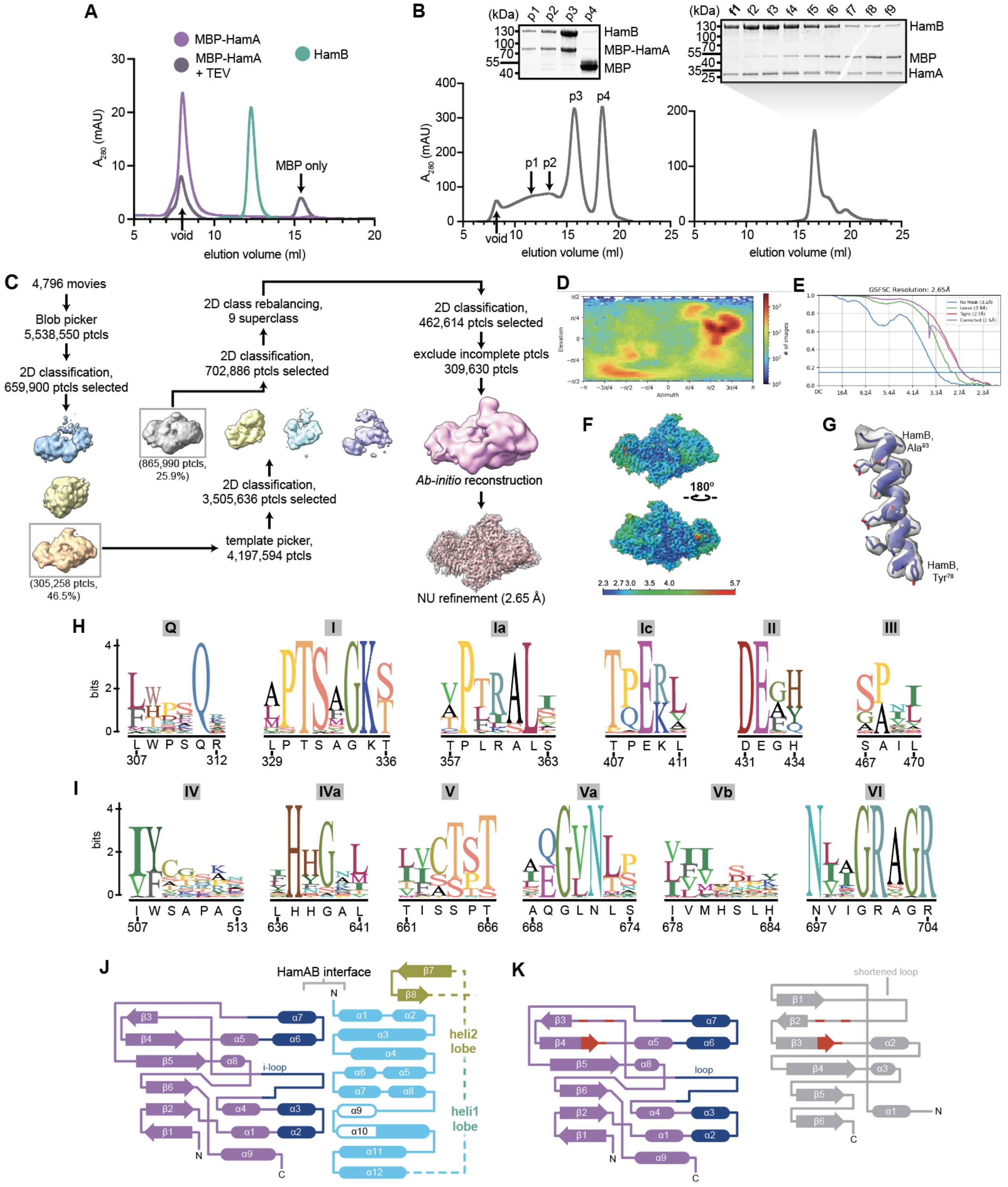

Figure S3 | Cryo-EM structure of the HamAB apo complex. (A) Size exclusion chromatography traces of MBP-HamA pre-TEV treatment, post-TEV treatment and HamB alone. **(B)** Left, size exclusion chromatography trace of MBP-HamAB and corresponding peaks run on a coomassie PAGE gel. Right, size exclusion chromatography trace of HamAB after TEV protease treatment, with elution fractions run on coomassie PAGE gel shown above. **(C)** Particle picking, classification, and refinement strategy to generate the final apo HamAB density. **(D)** Orientation distribution of the final particle set. **(E)** Gold standard FSC curve. **(F)** Sharpened map colored by local resolution. **(G)** Example model-to-map fit. **(H-I)** Sequence logos of helicase motifs in the RecA1 (H) and RecA2 (I) domains calculated from the HamB MSA. The residue number and identity of the corresponding sequence in ECOR31 HamB is shown below start and end motif positions. **(J)** Secondary structure diagram depicting the HamAB interaction interface. **(K)** Comparison of secondary structures of HamA and the *P. aquatile* Type IIS restriction endonuclease.

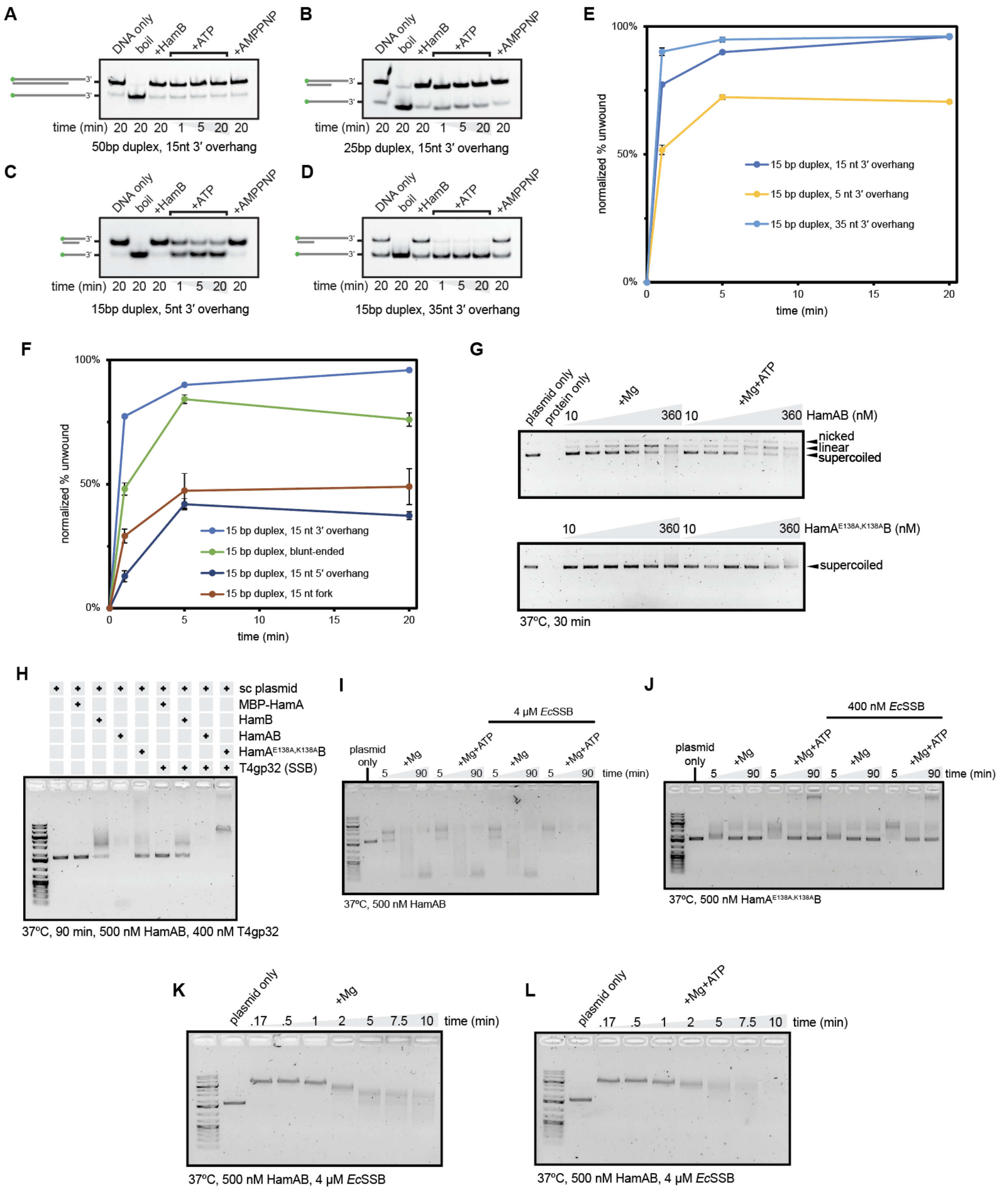

Figure S4 I Analysis of HamAB biochemical activities *in vitro*. **(A-D)** HamB DNA unwinding assays on substrates with a 50 bp duplex and a 15 nt 3′ OH (A), 15 bp duplex and 15 nt 3′ OH (B), 15 nt duplex with a 5 nt 3′ OH (D), and 15 nt duplex with a 35 nt 3′ OH (E). DNA substrates are labeled with 5′ FAM. Gels are representative of three independent biological replicates. **(E-F)** Normalized percent unwinding of DNA substrates with varying OH lengths (E) and different OHs (F). All substrates are labeled with 5' FAM. Shown are the mean and standard error of quantifications of three replications of unwinding assays in the format of A-D normalized against basal unwinding (see Methods). **(G)** Titration of HamAB WT and HamA*B complexes with plasmid DNA. **(H)** Comparison of Hachiman protein and complex plasmid activities with or without phage T4 SSB. **(I)** Time course of plasmid degradation by HamAB with or without ATP and saturating amounts of *E. coli* SSB. **(J)** Time course of plasmid degradation by HamA*B with or without ATP and limiting amounts of *E. coli* SSB. Conditions in absence of *E. coli* SSB are also shown in Fig. 3H. **(K)** Plasmid degradation over 10 mins with saturating *E. coli* SSB in the absence of ATP. **(L)** Plasmid degradation over 10 mins with saturating *E. coli* SSB and ATP supplied.

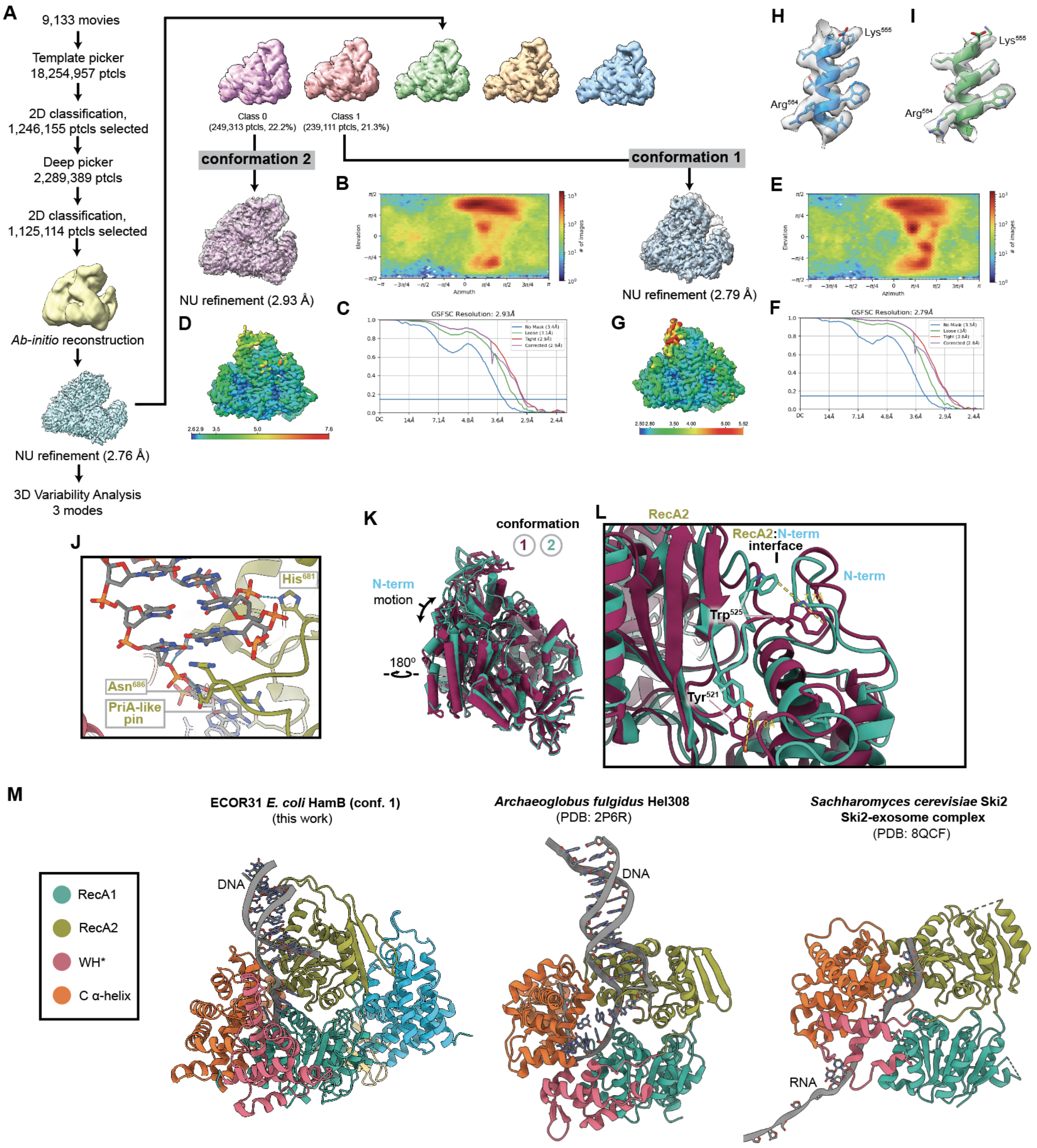

Figure S5 I Cryo-EM structure of two HamB-DNA complex conformations. **(A)** Particle picking, classification, and refinement strategy to generate the final HamB-DNA densities for conformations 1 and 2. **(B)** Orientation distribution of the final conformation 2 particle set. **(C)** Gold standard FSC curve for conformation 2. **(D)** Conformation 2 Sharpened map colored by local resolution. **(E)** Orientation distribution of the final conformation 1 particle set. **(F)** Gold standard FSC curve for conformation 1. **(G)** Conformation 1 sharpened map colored by local resolution. **(H)** Example model-to-map fit for conformation 2. **(I)** Example model-to-map fit for conformation 1. **(J)** Molecular detail of the PriA-like strand unwinding pin in HamB-DNA conformation 1. **(K)** Superimposition of conformations 1 and 2 viewed from the HamA side. **(L)** Detail of the HamB RecA2-NAH interface and comparison of conformational changes. **(M)** Comparison of HamB-DNA with other related helicases bound to their substrates. Middle, *A. flugidus* Hel308 (PDB: 2P6R). Right, Ski2-RNA from a structure of the *S. cerevisiae* Ski2-exosome complex (PDB: 8QCF). The exosome complex was hidden for clarity.

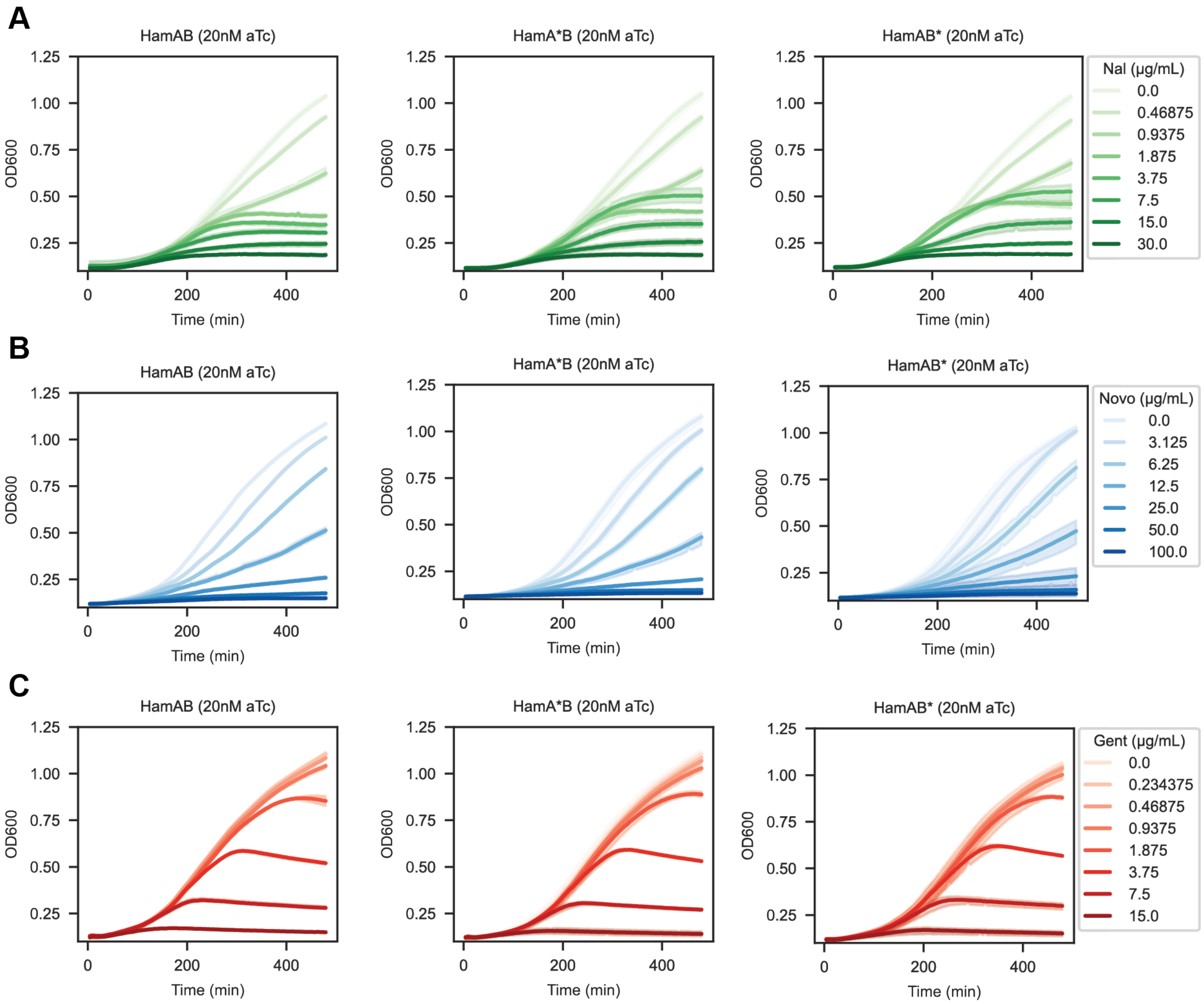

Figure S6 | Drug-induced DNA damage activates Hachiman. (A-C) Cell growth of *E. coli* expressing wildtype HamAB (left), nuclease-deficient HamA*B (middle) and helicase-deficient HamAB* (right) at 20nM aTc in the absence or presence of subinhibitory and inhibitory concentrations of nalidixic acid (A), novobiocin (B) or gentamycin (C). Growth curves are colored according to condition. All growth curves performed in biological triplicate.

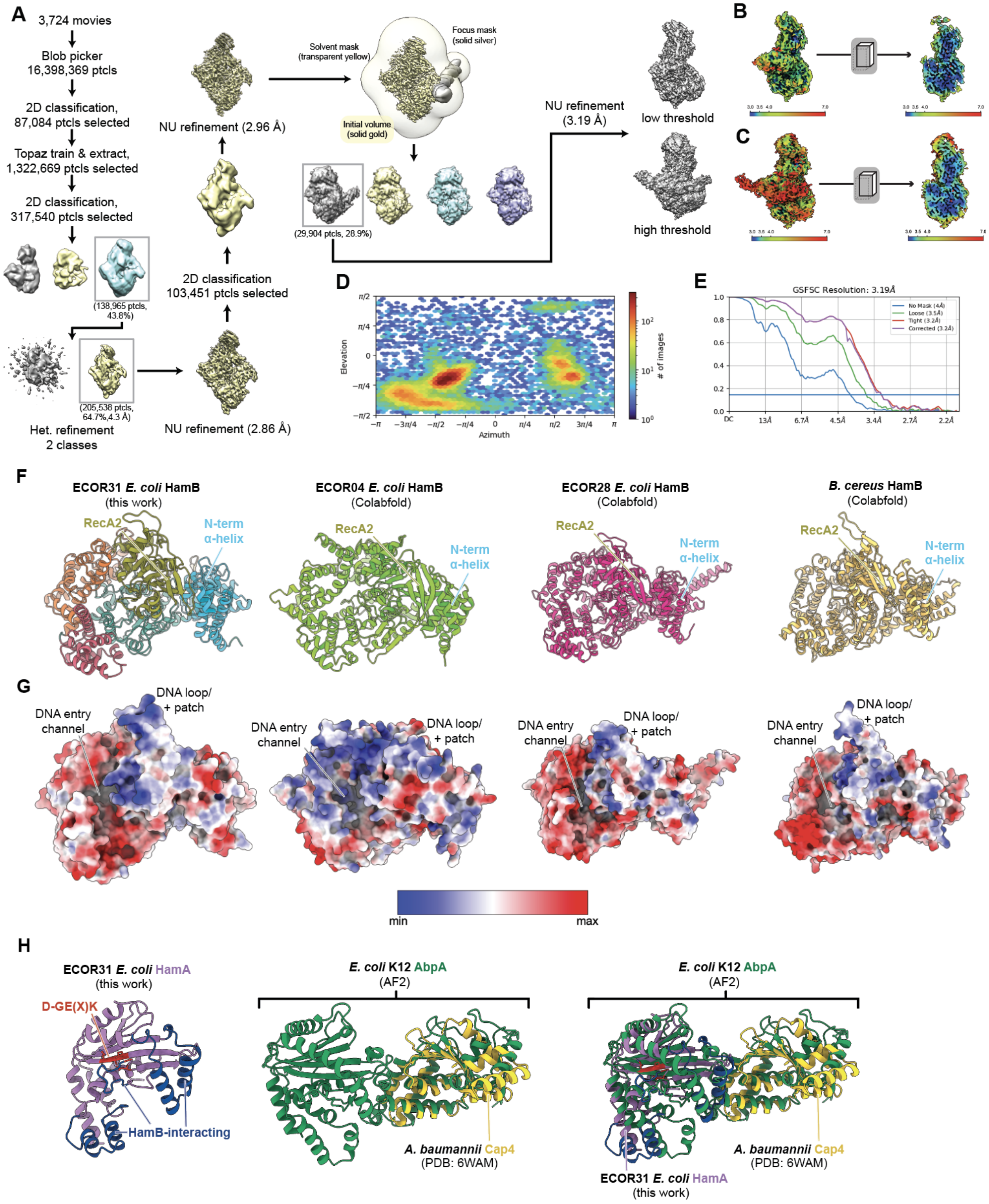

Figure S7 I Cryo-EM structure of a HamA(E138A, K140A)B-plasmid DNA complex. **(A)** Particle picking, classification, and refinement strategy to generate the final HamAE138A, K140AB-plasmid DNA density. **(B-C)** Final densities at low (B) and high (C) thresholds colored by local resolution. An inner surface slice is shown to the right. **(D)** Orientation distribution of the final particle set. **(E)** Gold-standard FSC curve. **(D)** Conformation 2 sharpened map colored by local resolution. **(E)** Orientation distribution of the final conformation 1 particle set. **(F)** Comparison of ECOR31 HamB from this study with ECOR04 HamB, ECOR28 HamB, and *Bacillus cereus* HamB Colabfold predictions. **(G)** Electrostatic surface potential representations of the structures from F, in the same orientation and scale. The DNA entry channel and RecA2 DNA loop/positively charged patch are indicated. **(H)** HamA and Cap4 structural superimpositions with AbpA.

Table S1 l Cryo-EM data collection, refinement, and validation statistics.

	E. coli ECOR31 apo HamAB (EMD-43613, PDB 8VX9)	*E. coli* ECOR31 HamB-DNA (conformation 1) (EMD-43615, PDB 8VXA)	*E. coli* ECOR31 HamB-DNA (conformation 2) (EMD-43616, PDB 8VXC)	*E. coli ECOR31* HamA(E138A,K140A)B-plasmid DNA (EMD-43643, PDB 8VXY)
**Data collection and processing**				
Voltage (kV)	300	200		300
Electron exposure (e^{-}/Å^{-2})	50	50		50
Defocus range (µm)	-0.8 to -1.8	-0.8 to -1.8		-0.8 to -1.8
Pixel size (Å)	1.05	1.12		1.05
Symmetry imposed	C1	C1		C1
Initial particle images (no.)	5,538,550	18,254,957		16,398,369
Final particle images (no.)	309,630	239,111	249,313	29,904
Map Resolution (Å)	2.65	2.79	2.93	3.19
FSC threshold	0.143	0.143	0.143	0.143
Map resolution range (Å)	2.3-5.7	2.5-4.8	2.6-7.6	2.9-7.0
**Refinement**				
Initial model used	AF2 model	AF2 model	8VXA (this work)	8VX9 (this work)
Model resolution (Å)	3.08	3.18	3.23	3.87
FSC threshold	0.5	0.5	0.5	0.5
Map-sharpening B-factor (Å^2)	-85.9	-92.5	-93.9	-60.9
Model composition				
Non-hydrogen atoms	11,244	9,431	9,391	12,324
Protein residues	1,433	1,139	1,139	1,406
Nucleotide residues	0	24	22	62
Ligands	0	0	0	1
B-factor (Å^2)				
Proteins	50.88	53.27	51.88	111.72
Nucleic acid	-	83.34	91.38	314.69
Ligands	-	-	-	89.85
R.M.S. deviations				
Bond lengths (Å)	0.27	0.003	0.003	0.003
Bond angles (°)	0.52	0.530	0.506	0.571
**Validation**				
MolProbity score	1.87	1.73	1.68	1.99
Clashscore	11.85	10.67	9.14	14.11
Poor rotamers (%)	0.08	0	0	0
Ramachandran plot				
Favored (%)	95.94	96.92	96.83	95.21
Allowed (%)	4.06	3.08	3.17	4.79
Disallowed (%)	0	0	0	0

Table S2 l Strains used in this study

**Strain**	**Plasmid**	**Use**
E.coli dh10b	N/A	Phage assays, antibiotic sensitivity assays, microscopy, cloning
E.coli BL21-AI	N/A	Protein expression and purification
E.coli BW25113	N/A	Phage propagation
E.coli dh5a F'	F'	Propagation and assaying phages MS2 and M13
E.coli DSM103255	N/A	Propagation of phage G17
E.coli MC1000	N/A	Propagation and assaying phage Goslar
E.coli ECOR47	N/A	Assaying phage G17
E.coli ECOR04	N/A	Wildtype locus of ECOR04 Hachiman
E.coli ECOR28	N/A	Wildtype locus of ECOR28 Hachiman
E.coli ECOR31	N/A	Wildtype locus of ECOR31 Hachiman

Table S3 | Plasmids used in this study

Plasmid Number	Description	Origin	Resistance	Use	Source
pBA635_ddCasRX_RFP_CDS_1	Catalytically deactivated RfxCas13d under pTet control (using RFP-targeting guide)	p15a	Cm	Negative control for plaque assays.	[88]
pBA1368_pTet-ECOR04_Hachiman	Hachiman from ECOR04 under pTet control.	p15a	Cm	Bacterial assays for ECOR04 Hachiman including phage infection assays.	Current study
pBA1369_pTet-ECOR28_Hachiman	Hachiman from ECOR28 under pTet control.	p15a	Cm	Bacterial assays for ECOR28 Hachiman including phage infection assays.	Current study
pBA1370_pTet-ECOR31_Hachiman	Hachiman from ECOR31 under pTet control.	p15a	Cm	Bacterial assays for ECOR31 Hachiman including phage infection assays and antibiotic sensitivity assays.	Current study
pBA1444_ECOR31HamA_purification	MBP-His-tagged ECOR31 HamA for protein purification	ColE1	Carb	Protein expression and purification of ECOR31 HamA.	Current study
pBA1445_ECOR31HamB_purification	MBP-His-tagged ECOR31 HamB for protein purification	ColE1	Carb	Protein expression and purification of ECOR31 HamB.	Current study
pBA1464_pTet-ECOR31Ham_DELhamA_Mut	Hachiman from ECOR31 under pTet control with HamA deleted (and HamB native RBS intact).	p15a	Cm	Bacterial assays for mutant ECOR31 Hachiman including phage infection assays. Mutant encodes for a HamA deletion, leaving the HamB RBS intact.	Current study
pBA1465_pTet-ECOR31Ham_DELhamB_Mut	Hachiman from ECOR31 under pTet control with HamB deleted.	p15a	Cm	Bacterial assays for mutant ECOR31 Hachiman including phage infection assays. Mutant encodes for a HamB deletion.	Current study
pBA1467_pTet-ECOR31Ham_hamB_walkerB_Mut	Hachiman from ECOR31 under pTet control with HamB mutated (D431A).	p15a	Cm	Bacterial assays for mutant ECOR31 Hachiman including phage infection assays and antibiotic sensitivity assays. Mutant encodes for a HamB helicase active site mutant.	Current study
pBA1468_pTet-ECOR31Ham_hamA_D119A_Mut	Hachiman from ECOR31 under pTet control with HamA mutated (D119A).	p15a	Cm	Bacterial assays for mutant ECOR31 Hachiman including phage infection assays. Mutant encodes for a HamA nuclease active site mutant.	Current study
pBA1469_pTet-ECOR31Ham_hamA_E138A_K140A_Mut	Hachiman from ECOR31 under pTet control with HamA mutated (E138AK140A).	p15a	Cm	Bacterial assays for mutant ECOR31 Hachiman including phage infection assays and antibiotic sensitivity assays. Mutant encodes for a HamA nuclease active site double mutant.	Current study
pBA1580_ECOR31HamAB_CoPurification	MBP-His-tagged ECOR31 HamA operonic with HamB for protein purification	ColE1	Carb	Protein expression and purification of ECOR31 HamAB complex.	Current study
pBA1583_ECOR31HamA(E138AK140A)B_CoPurification	MBP-His-tagged ECOR31 mutant HamA (E138AK140A) operonic with HamB for protein purification	ColE1	Carb	Protein expression and purification of mutant ECOR31 HamAB complex. Mutant encodes for a HamA nuclease active site mutant. Mutant encodes for a HamA nuclease active site double mutant.	Current study
pBA1606_pTet-ECOR31HamAB_Mut_HamAΔdza 166-195	Hachiman from ECOR31 under pTet control with HamA mutated (Δ166-195).	p15a	Cm	Bacterial assays for mutant ECOR31 Hachiman including phage infection assays. Mutant encodes for a HamAB interface mutant in HamA.	Current study
pBA1607_pTet-ECOR31HamAB_Mut_HamA_mut3 RXXK_AXXA (104, 108)	Hachiman from ECOR31 under pTet control with HamA mutated (R104A, K108A).	p15a	Cm	Bacterial assays for mutant ECOR31 Hachiman including phage infection assays. Mutant encodes for a HamAB interface mutant in HamA.	Current study

Table S4 | Phages used in this study

Phage	Propagation Host	Assay Host	Class (ICTV 2022)	Genus (ICTV 2022)	Culture Notes
EdH4	*E.coli* BW25113	*E.coli* BW25113	Caudoviricetes	Vequintavirus	
G17	DSM103255	*E.coli* ECOR47	Caudoviricetes	Asteriusvirus	Plaque assays require 0.375% top agar
Goslar	*E.coli* MC1000	*E.coli* MC1000	Caudoviricetes	Goslarvirus	Plaque assays require 0.375% top agar
M13	*E.coli* Dh5a F'	*E.coli* Dh5a F'	Faserviricetes	Inovirus	Add 1mM CaCl2, Need F' plasmid
MM02	*E.coli* BW25113	*E.coli* BW25113	Caudoviricetes	Mosigvirus	
MS2	*E.coli* Dh5a F'	*E.coli* Dh5a F'	Leviviricetes	Emesvirus	Add 1mM CaCl2, Need F' plasmid
N4	*E.coli* BW25113	*E.coli* BW25113	Caudoviricetes	Enquatrovirus	
PTXU 04	*E.coli* BW25113	*E.coli* BW25113	Caudoviricetes	Xuquatrovirus	
SUSP 1	*E.coli* BW25113	*E.coli* BW25113	Caudoviricetes	Suspvirus	
T4	*E.coli* BW25113	*E.coli* BW25113	Caudoviricetes	Tequatrovirus	
T5	*E.coli* BW25113	*E.coli* BW25113	Caudoviricetes	Tequintavirus	
T7	*E.coli* BW25113	*E.coli* BW25113	Caudoviricetes	Teseptimavirus	

Table S5 | ATPase assay oligonucleotide substrates

Number	Substrate Descrip-tion in Figure 3A	Sequence (5' to 3', r prefix represents RNA)	sequence length (nt)
1	polyT ssDNA	TTTTTTTTTTTTTTTTTTTTTTTTTTTTTTTTTTTTTTTT	40
2	mixed ssDNA	TCGTCACCAGTACAAACTACAACGCCTGTAGCATTCCACA	40
3	5' overhang DNA	TCGTCACCAGTACAAAC	17
		TTTTTTTTTTTTTTTTGTTTGTACTGGTGACGA	33
4	3' overhang DNA	TCGTCACCAGTACAAAC	17
		GTTTGTACTGGTGACGATTTTTTTTTTTTTTTT	33
5	complete duplex DNA	TCGTCACCAGTACAAAC	17
		GTTTGTACTGGTGACGA	17
6	polyA ssRNA	rA-<br>rArArArArArArArArArArArArArArArArArArArArArA<br>rArArArArArArArArArArArArArArArArArArA	40
7	mixed ssRNA	rUrCrGrUrCrArCrCrArGrUrArCrArArArCrU-<br>rArCrArArCrGrCrCrUrGrU-<br>rArGrCrArUrUrCrCrArCrA	40
8	5' overhang RNA	rUrCrGrUrCrArCrCrArGrUrArCrArArArC	17
		rU-<br>rUrUrUrUrUrUrUrUrUrUrUrUrUrUrUrGrUrUrUrGrU<br>rArCrUrGrGrUrGrArCrGrA	33
9	3' overhang RNA	rUrCrGrUrCrArCrCrArGrUrArCrArArArC	17
		rGrUrUrUrGrUrArCrUrGrGrUrGrArCr-<br>GrArUrUrUrUrUrUrUrUrUrUrUrUrUrUrUrU	33
10	dsRNA	rUrCrGrUrCrArCrCrArGrUrArCrArArArC	17
		rGrUrUrUrGrUrArCrUrGrGrUrGrArCrGrA	17
11	RNA/DNA hybrid w/ 3' DNA overhang	GTTTGTACTGGTGACGATTTTTTTTTTTTTTTT	33
		rUrCrGrUrCrArCrCrArGrUrArCrArArArC	17
12	DNA/RNA hybrid w/ 3' RNA overhang	TCGTCACCAGTACAAAC	17
		rGrUrUrUrGrUrArCrUrGrGrUrGrArCr-<br>GrArUrUrUrUrUrUrUrUrUrUrUrUrUrUrUrU	33
13	blunt dsDNA/RNA hybrid	TCGTCACCAGTACAAAC	17
		rGrUrUrUrGrUrArCrUrGrGrUrGrArCrGrA	17

Table S6 | Unwinding assay oligonucleotide substrates

Number	Substrate Description	Sequence (5' to 3', * represents 6-FAM)	sequence length (nt)
1	15bp duplex, with 15nt 3' overhang	*ATATCGTAGGTATGGTGGAGGCCGGTAGGT	30
		CCATACCTACGATAT	15
2	25bp duplex, with 15nt 3' overhang	*ATATCGTAGGTATGGTGGAGGCCGG-TAGGTAATTCTAGCC	40
		CCGGCCTCCACCATACCTACGATAT	25
3	50bp duplex, with 15nt 3' overhang	*ATATCGTAGGTATGGTGGAGGCCGG-TAGGTAATTCTAGCCCAGCTCCATGCGAGCACTAC-CAATC	65
		CATGGAGCTGGGCTAGAATTACCTACCGGCCTCCAC-CATACCTACGATAT	50
4	15bp duplex, with 5nt 3' overhang	*ATATCGTAGGTATGGTGGAG	20
		CCATACCTACGATAT	15
5	15bp duplex, with 35nt 3' overhang	*ATATCGTAGGTATGGTGGAGGCCGG-TAGGTAATTCTAGCCCAGCTCGATG	50
		CCATACCTACGATAT	15
6	15bp duplex, blunt-ended	*ATATCGTAGGTATGG	15
		CCATACCTACGATAT	15
7	15bp duplex, with 15nt 5' overhang	*ATATCGTAGGTATGGTGGAGGCCGGTAGGT	30
		ACCTACCGGCCTCCA	15
8	15bp duplex, with 15nt fork	*ATATCGTAGGTATGGATCATTGGCGGATGA	30
		GGTCATTAGTCCTCGCCATACCTACGATAT	30
